# Intrinsic social media literacy: a public relations strategy addressing problematic social media use among teenagers globally

**DOI:** 10.1080/23311886.2025.2518437

**Published:** 2025-06-13

**Authors:** Funom Theophilus Makama, Stephanie Attah James, Esther Funom Makama

**Affiliations:** a Human Rights & Global Ethics, School of History, Politics and International Relations, University of Leicester, Leicester, UK; b Public Health (International) Nuffield Centre for International Health and Development, Leeds Institute of Health Sciences, University of Leeds, Leeds, West Yorkshire, UK; c Public Relations and Strategic Communications, Centre of Public Relations, Leeds Beckett University, Leeds, UK; d Member of Management Team, Brushstrokes Community Project, Birmingham, UK

**Keywords:** Problematic social media use, social media addiction, public relations strategy, social media literacy, adolescent use of social media, parental control of social media, social media control applications, Media Communication, Public Relations, Visual Communication, Persuasion

## Abstract

On average, 6 hours 58 minutes are spent on social media everyday by each user, and 98% of young people from ages 15 to 24 use the internet of which 96% are social media users. If used inappropriately, a social media user is at risk of poor mental health and behavioural problems. Also, Problematic social media users are vulnerable to several side effects and are more exposed to social media threats than any other user. Therefore, this study attempts to develop a preventive strategy by firstly, analysing the various approaches that address problematic social media use such as the legal, education and technology strategies. By assessing their strengths and weaknesses, the most effective approach- Social Media Literacy was identified. The second objective of this study is to address the weaknesses of all the approaches analysed, and by using public relations mechanisms, social media literacy was enhanced into a new approach called the Intrinsic Social Media Literacy, ISML. This new approach is not developed to stop other preventive approaches from addressing this issue. It is developed as the main approach in the prevention, and to some degree, the solving of problematic social media use among the young generation globally.

## Introduction

### Background

There are approximately 2.9 billion, and 1.4 billion monthly users of the Meta-company-owned social media platforms- Facebook and Instagram respectively (Daniel, 2022). Also, 98% of young people from ages 15 to 24 use the internet daily (European Commission, 2019) and 96% of them use social media, mostly sharing opinions, news and experiences. Whilst 35% of them say it will be “somewhat” hard for them to stop using social media, 18% admitted that it would be “hard,” while a whopping 54% say it would be “very hard” to stop using social media (European Commission, 2019). In another study of 1,316 American teenagers that are aged 13 to 17, the findings show that the most frequently used social media are Tiktok, Instagram, and Snapchat (Lenhart, 2015). Currently, YouTube, Tiktok and Snapchat are leading in the “popularity contest” of social media usage among the young population as 67% of teenagers use TikTok while Snapchat has a massive increase in usage from 41% in 2015 to 59% in 2022. The only social media platform that is more popular among teenagers than Tiktok is YouTube, which enjoys a massive 95% usage from the same population. Facebook, being a “conventional social media platform” in terms of more restrictions and regulations has suffered a decline from 71% in 2015 to 32% in 2022 among the young population (Taibi et al., 2022, p. 316). Excessive screen time or digital use by adolescents has been rated among the Australian public in 2015 as the most worrisome health concern even ahead of obesity, child abuse and suicide and this concern quickly spread fears across the globe. Since excessive digital use increases the vulnerability of adolescents to become problematic users, especially that of social media, something needed to be done to address it (Festl, 2021, p. 250). These statistics mean that the young are exposed to highly heterogenous and harmful contents (Fulantelli et al., 2022).

### Definitions

1. *Teenagers:* Specifically, to this research, teenagers are a demographic of young people from 13 to 19. Sometimes, “young population” may be used across this study, which also align to this definition (Abd Rahman and Abdul Razak, 2019; Rahman et al. 2019).
2. *Social media:* A developed Application that can be used on phones or computers which creates the forum for the sharing of ideas, feelings and activities within the network of connecting people. This usually needs the internet to function (Abd Rahman and Abdul Razak 2019; Rahman et al. 2019). It is the broad group of third-party internet-based platforms that provide the non-physical or virtual avenue for social interactions, community-based inputs and content sharing among community users who participate in the same platforms. The major characteristic of these third-party platforms is that they only feature contents that are created by their registered or subscribed users. They are also known as social networking sites and examples are Facebook, YouTube, WhatsApp, Instagram, WeChat, TikTok, Telegram, Snapchat etc (Pellegrino et al., 2022, pp. 1–2). Therefore, social media usage is of huge concern in this generation as, for instance, the average American scrolls about 305 feet length of social media pages every day on Facebook alone. This is significant because the statue of liberty is about 305 feet tall (Daniel, 2022); which is also equivalent to the length of 44 basketball players put together at a national average of 6 feet, 6 inches per player (The Hoops Geek, 2022). This is the length of pages scrolled everyday by an average American user of Facebook.
3. *Addiction:* This is a behavioural abnormality which is characterized by the inability to consistently abstain from an activity or a material thing. Features such as cravings, changes in behavioural pattern, poor recognition of problems with one’s interpersonal relationships and behaviours and dysfunctional emotional response are characteristic symptoms of addiction. It also often involves the cycles of relapse and remission. Using the term social media addiction is contentious within the fields of mental health and human behavioural science as would be discussed next (Brand et al., 2022; Pellegrino et al., 2022).
4. *Problematic Social Media Use (PSMU):* this is best defined as a non-substance-related disorder that results in the preoccupation and compulsion to engage excessively in social media platforms despite negative consequences (Andreassen et al., 2017, p. 287). Some signs of PSMU include spending excessive time on social media, neglecting other activities in favour of social media, frequently arguing about social media use, lying about how much time is spent on social media and experiencing withdrawal from social media (Cataldo et al., 2022, p. 101145). PSMU may lead to any of the following negative outcomes: low self-esteem, body dissatisfaction, higher levels of substance use etc (Paakkari et al., 2021). Social media addiction is usually associated with one of, some or all three personality traits: anxiety, depression and extraversion. Minor traits associated with social media addiction include narcissism, rise in stress levels, sadness, physical problems such as obesity and carpel tunnel syndrome and other problems such as financial mismanagement and low academic performance in school (Pellegrino et al., 2022, p. 3). But social media addiction is a diagnosis and a terminology that should be backed with diagnostic criteria, which unfortunately, have not been satisfactorily established due to the lack of high-quality long-term studies. Moreso, there are people who use social media the problematically, affecting the social, emotional and mental aspects of their lives without reaching the limits of “anxiety”, “depression” or “extraversion”. Also, not all excessive social media users are narcissists. For instance, young users may develop low self-esteem for constantly comparing themselves, their appearances or lifestyles with other users they see or subscribe to, on social media platforms. The low self-esteem developed is not as a result of “addiction” but as a result of their excessive usage of the platform. It is for this reason that the terminology “problematic social media use”, PSMU, is generally preferred, and though, many scholars do not classify social media disorders or addiction as PSMU (Paakkari et al., 2021, p. 1), for the sake of this study, they will all be classified as PSMU. Also, since Leena Paakkari and colleagues (2022) argue in their study that PSMU also leads to anxiety, poor mental health and depression, PSMU will therefore be used as an umbrella terminology to cover a broad spectrum of the negative usage of social media from being sad because of its excessive use to the exhibition of symptoms of addiction (Brand et al., 2022).

### Increased social media usage during the pandemic

COVID-19 pandemic influenced a surge in social media usage worldwide. In India for instance, there was an 87% increase in the number of people using social media and a 75% increase in the time spent on Facebook, Twitter and WhatsApp (Singh et al., 2020). Though, it started as a coping mechanism to deal with the pandemic, subsequently, the trend was highly suggestive of problematic social media use as indicated by the observable increase in anxiety, depression and other associated conditions such as poor cardio-metabolic health, poor sleep, affect, low self-esteem, poor well-being and functioning, especially in adolescents (Gao et al., 2020; Tandon, 2020). During the pandemic, changes in social circumstances such as social distancing and the loss of intimate interpersonal contact alongside restrictions such as lockdowns, strict hygienic routines and protocols etc., were already causing increased incidences of poor mental health conditions. Different parts of the world reported increased incidences of anxiety, depression, the feeling of frustration and boredom (Brooks et al., 2020). But the pandemic also influenced the young population globally, mostly teenagers and college students to rely on social media to relieve the negative aspects of isolation (Liu et al., 2020, p. 17). But since the lockdowns ensured that they stayed at home for long periods, their use of social media became excessive which led to their sustained exposure of excessive information, misinformation, threats and side effects of prolonged use of social media and by the time the pandemic was over, a significant portion of this young population became problematic social media users (Daniels et al., 2021). Youngrong Lee and colleagues (2022) did a meta-analysis of fourteen studies to find out the association between the increase in the time spent on social media networking sites during the pandemic and poor mental health symptoms. They concluded that the excessive time spent on social media during COVID-19 is linked to a greater likelihood of having symptoms of anxiety and depression.

### The public relations strategy

Public relations efforts/strategies/mechanisms/actions are communications activities that propagate and enhance the visibility and the sculpt public perceptions of a situation or image and affect a change of these perceptions. These efforts are usually to create a positive public image for business, non-profit organisations and individuals. These efforts, strategies, mechanisms or actions are usually termed “initiatives” or “campaigns”. But with the change in time from traditional methodologies to the modern digital media era, the propagation, functionality and objectives of public relations have also evolved from its nature of conversations, purpose, reach, effect and even its suitability to cultures (Erzikova et al., 2018). Thus, developing effective messages for the dissemination of information to strategically important publics is even more critical currently. It is, hence, paramount for theorists and public relations professionals to develop message strategies for communicating with the public to reach strategic goals (Werder & Holtzhausen, 2009, p. 408). Seven strategies are commonly used in public relations by organizations to attain their goals with the target public they intend to interact with. These strategies are, (1) informative, (2) persuasive, (3) facilitative, (4) cooperative problem solving, (5) promise and reward, (6) threat and punishment, and (7) bargaining (Hazleton, 1992, 1993; Werder, 2005). Therefore, public relations is so advanced in current times that its aim is beyond improving the image of a brand. Public relations tools, mechanism or strategies can be adopted to solve problems as well (Rahim, Markom and Alsagoff 2019), and one of which is the focus of this study- PSMU. As stated by Werder and Holtzhausen (2009, p. 410-11), there are five operational strategies which enable the effectiveness of public relations in achieving goals by an organization and Table 1 below illustrates them.

**Table 1.** Operational strategies of public relations.

Strategies	Definitions
Informative	To present unbiased facts. Informative messages do not draw conclusions but presume that the target public can discern for themselves to deduce appropriate conclusions from accurate data. Informative messages may point towards a range of options that may solve problems and they typically use neutral language. The use of Mass media is the most likely and ideal approach in adopting this strategy by an organization. Though, when “neutral messaging” is not used in this strategy, the Mass Media mechanism becomes dangerous as would be discussed later.
Facilitative	To make available, resources to the public, which will influence the members of the target group to act in ways that they are already predisposed to act. Resources may be tangible artifacts, such as tools or money; they could also be directions that tell someone how to accomplish a particular action e.g. directional billboard. They could also be Artificial intelligent tools that create awareness to help identify fake news, toxic contents, discriminatory materials or contents highly concentrated with an Agenda etc.
Persuasive	To appeal to the public’s values or emotions. This strategy is characterized by the selective presentation of information, using bias language, which is not neutral due to the importance of the issue and the organisation’s involvement in the situation. Persuasive messages provide a call for action, either directly or indirectly. The mass media strategy may still fall under this category, though not ideal. Community engagements, public health approaches and the use of law and policies may adopt this strategy in solving problems like PSMU.
Cooperative problem-solving	To identify and solve problems as groups, societies, members of an organization or team through the “open exchange of information” mechanism. But common goals and shared responsibilities about the situation must be established for this strategy to work. It is also paramount to note that the messages disseminated must create the sense of interdependence between an organization and its public and the use of inclusive language such as “we” are essential to the effectiveness of this strategy. Public health approaches and community engagement mechanisms suit this strategy.
Power	To exercise power to gain compliance directly or indirectly. This strategy is implemented in two forms: promise and reward strategies and then, threat and punishment strategies. In the promise and reward component, the organization in charge is in control of the public’s desired outcome. But in the “threat and punishment” methodology, the organization in charge is in control of an outcome feared or disliked by the public. In this case, the use of law to tackle social media addiction is well-suited.

Source: Adopted and modified from Werder and Holzhausen 2009.

### Problem statement

**Figure 1.**
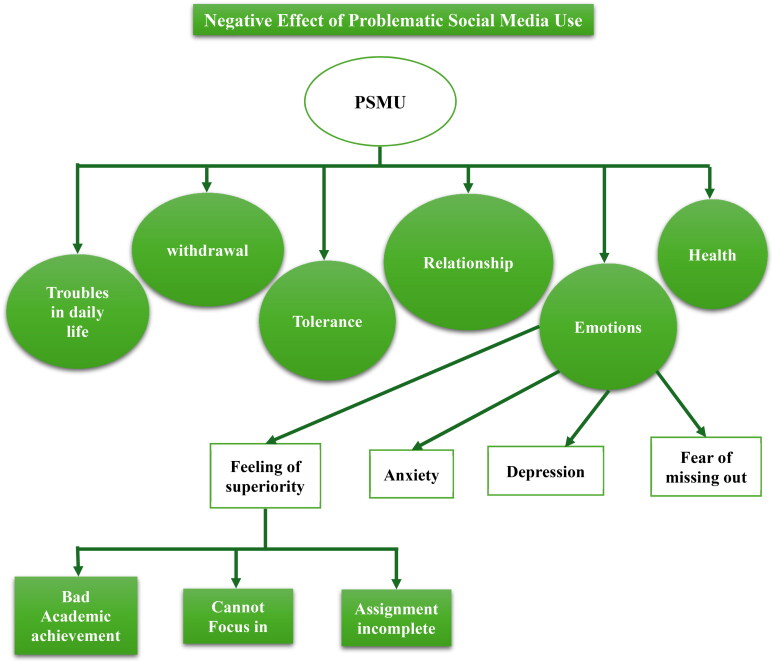
Negative effects of Problematic Social Media Use. Adopted from the theoretical framework of Abd Rahman and Abdul Razak’s paper (2019, p. 4).

The world’s population was reported to be about 7.91 billion people as of 2022 and 4.62 billion were reported to be active social media users, averaging 6 hours, 58 minutes spent per day per person on social media. If used inappropriately, a user is at risk of poor mental health and there are evidence linking social media usage to suicidality, anxiety and loneliness. In fact, the terminology “Facebook depression” was created by the American Academy of Paediatrics, AAP, which is a condition of depression exhibited by preteens and teens after spending too much time on social media platforms such as Facebook (Pellegrino et al., 2022, p. 2). Another study carried out on 11-, 13- and 15-year-olds in 45 countries gave findings that showed 35% of respondents to be characterized as “intensive electronic media users” while about 7% were considered problematic social media users (Paakkari et al., 2021, p. 1). PSMU has also been reported in many studies to have increased in the past decade as of the time of writing this review (Brand et al., 2022, p. 3-4). In the USA, teenagers of ages 13 to 17 use Facebook the most and 71% of teenage social media users access more than one social media networking site while 24% of adolescents are almost always online (Bányai et al., 2017, p. 1). Therefore, PSMU is of huge global concern and particular interest should be given to teenagers of ages 13 to 19, which is the focus of this study. Severe PSMU with symptoms of addiction, just like shopping, persistent gambling or exercise addiction, is classified as a non-chemical addiction, different from the chemical type such as substance abuse. Non-chemical addictions have seven core symptoms in common and they are: Salience, tolerance, mood modification, conflict, withdrawal problems and relapse (Grant et al., 2010; Griffiths, 2005). Also, young people who are problematic social media users are prone to the “co-addiction” of other related digital activities such as online video games, internet-enabled devices and general smart phone addiction in what is generally termed “Digital Addiction” (Almourad et al. 2020; Brand et al., 2014). PSMU also causes reduced wellbeing, emotional problems even to severe degrees such as severe depressive symptoms and suicidal thoughts (Taibi et al., 2022, p. 316). Figure 1 below summarises the negative effect of PSMU. And with the increasingly high prevalence rate of PSMU around the globe, Tim Stanley (2018) in his article in The Telegraph termed PSMU as a “public health emergency” which he feared to be killing us all.

Therefore, PSMU is a serious issue of grave concern that must be tackled urgently. For this to be effective, an intervention is needed to prevent and possibly solve this problem. One that requires an approach that will increase the critical thinking ability among the young population as it develops their self-protection skills, emotional intelligence and empathy, all driven by intrinsic motivation (Taibi et al., 2022, p. 316-317). Therefore, the scope of this study will cover the side effects of PSMU, the threats from social media in general and then the possible interventions.

#### Side effects

As stated earlier, PSMU may result to salience, mood modification, withdrawal, relapse, conflict and other problems. As defined by Cecilie Schou Andreassen (2015, p. 176), the various conditions mentioned below are side effects of PSMU and they are:

*Salience:* This is spending too much time on social media and when away, thinking of how to return to it.
*Mood modification:* In this case, the problematic social media user uses social media as a comfort zone, as it is perceived to be a medium to reduce anxiety, guilt, helplessness, restlessness and even depression. Social media is used to forget personal problems.
*Withdrawal:* When not allowed to use social media, either through prohibition or unavailability, problematic social media users typically become stressed, irritable, troubled and restless.
*Relapse:* For those trying to cut down on the excessive usage of social media, it usually does not take long for them to return to their usual social media activities.
*Conflict:* problematic social media users give less priority to other functions or events of their lives such as a job, hobby, studies, exercise, leisure activities etc. It can even get worse, affecting their relationships with their partners, family members, friends, colleagues etc.
*Problems:* PSMU has a direct negative impact on health- mental wellbeing especially. It also negatively affects sleep quality and as already mentioned, relationships.

#### Threats

There are threats that a social media user is exposed to. This means that a problematic user is much more exposed to these threats than a “regular” user. Some of the threats are highlighted below.

*Filter bubbles:* These are algorithms used by search engines, social media platforms and other large online networking platforms to reduce the diversity of information, viewpoints, perspectives, opinions and ideas that supposed to be readily available online. This threat easily leads to manipulation and misinformation online (Bozdag & Van Den Hoven, 2015). A problematic user is more exposed to this threat than a regular social media user.
*Echo Chambers:* Homophily is the tendency for a person to only network with others of similar views and perspective of life. This is unfortunately, the guiding principle of most social media platforms. Homophily may amplify bigotry and a “tribal perspective” that may lead to the degradation of the quality, safety and diversity of an online discourse, hence known as echo chambers (Gillani et al., 2018).
*Digital Wildfires:* The ability to spontaneously post and repost digital content is one huge benefit of social media. Unfortunately, this can be a nutritive ground for rumour, false and malicious spread of information., This is termed by Helena Webb and her colleagues (Webb et al., 2016) as digital wildfires. A problematic user may be caught up with this “trap.”
*Impulsivity:* This is the tendency to act without thinking about consequences and as much as it is considered a character trait for some people, it is a symptom of a medical disorder to others (Rudy, 2023). Impulsivity has been proven to be an addictive behaviour to problematic social media users and other addicts of gambling, tobacco use, opioid use, methamphetamine, the ecstasy drug, 3,4-methylene­dioxymethamiphetamine or simply MDMA, cocaine, cannabis and alcohol (Lee et al., 2019).
*Confirmation bias:* When a particular belief or perspective is heavily shared so that proponents of that belief conveniently ignore dissenting information, it becomes confirmation bias. The wide availability of similar content, which is common with social media platforms, fosters the aggregation of like-minded people where debates fuel group polarization (Del Vicario et al., 2017).
*Social reinforcement:* This is the feedback as exhibited in actions such as praise, acclaim, acceptance, attention and smiles. As for social media, the use of “emojis,” “comments,” “reposting and postings,” “smileys” etc, are tools of reinforcement. They can either encourage or discourage individuals from engaging in a behaviour. These reinforcements have psychological effects on humans as would be discussed later and social media companies take advantage of them to create and sustain an addiction mechanism on their young customers. When a bad behaviour is encouraged, a social reinforcement occurs. This is also a threat that problematic users should not be exposed to Cherry (2022; Lundahl, 2021, p. 1107).
*Backfire Effect:* As much as social media exacerbates political polarization, leading to “echo chambers,” it also causes a “backfire effect.” Some studies conducted over liberals and conservatives showed that liberals became even more liberal after being exposed to some conservative views on social media and conservatives became even more conservative after being exposed to some liberal views on the same platform, particularly twitter (Bail et al., 2018).
*Emotional Load:* Some moral and political ideas spread more rapidly than others on social media platforms such as twitter, which among a few others are believed to have altered the course of some historic events from the Arab spring to US presidential elections. In this regard, social media can negatively lead to emotional load on problematic users (Brady et al., 2017). More moral-emotional language can be found in political messages with themes such as same-sex marriage, gun control, climate change, etc (Hess & Fischer, 2013; Schier & Eberly, 2016).
*Anonymity & Deindividuation:* Social media platforms are forums that allow for anonymity. A functional account can carry identities that do not match the person using these accounts and this may pose serious threat to the real world. A problematic user could either be prone to take advantage of this Anonymity or be more susceptible to becoming a victim (Ureña et al., 2019). When this anonymity, together with self-awareness is being manipulated to cause the transgression of general social norms, it is called deindividuation, and this is a typical side effect of social media which problematic users may be more exposed to Postmes and Spears (1998).

By showing the worrying global trends of PSMU, problematic social media use has been identified as a significant global problem. The negative effects and threats which problematic social media users are exposed to gives the objective view of why PSMU should be of concern. Therefore, the next steps to this study is to identify the strategies in place that are tailored towards addressing PSMU among the young population globally, and to evaluate or develop the most effective strategy that will address it. This leads us to the research questions of this study

## Research questions

1. Is problematic social media use a significant global problem?
2. Is there the urgent need to address problematic social media use globally?
3. What are the strategies in place that are tailored towards addressing problematic social media use among the young population globally?
4. What is the most effective way of addressing problematic social medial use in the young population globally?

By addressing these questions, the aim of this study would be achieved.

### Aim of study

To identify the ongoing approaches that address problematic social media use and critically analyse them to develop the most effective strategy that will address the issue globally.

## Materials & methods

### Conceptual framework

**Figure 2.**
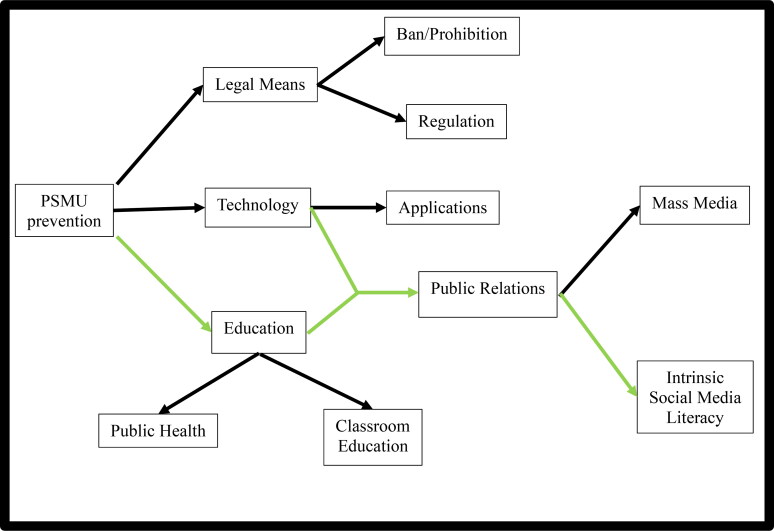
The Conceptual Framework for this Research. Source: Authors.

The conceptual framework of this study demonstrates the research scope and how the central theme of study will be analysed. The negative effect of PSMU will be discussed and interventions analysed into three different ways, the legal means, the technological means and the educational means. The legal means involves legality and legislation such as banning of social media for teenagers to gain access. It could also mean developing a control mechanism by the government or government-sponsored body to regulate the use of social media by teenagers, The technological means involves the use of phone applications to reduce the amount of time spent on social media. The educational means can be in two categories, public health interventions through health promotion, epidemiology and radical interventions such as counselling, or education in the classroom as part of an academic curriculum. Technology may enhance the education of social media to prevent or stop addiction, and this will require some public relations strategies. This is achieved via mass media or social media literacy by teachers or educators. Figure 2 below shows this framework, and the green arrows indicate the focus of this study.

### Research design

This study will follow the pattern as shown in Table 2 below. By addressing the research questions, the research objectives would be met, and the outcomes of these objectives will lead to the clear understanding of the problem, its implications and how to best address it.

**Table 2.** Research design.

Research question	Research objectives	Methods	Outcome
AIM: To identify the ongoing approaches that address problematic social media use and critically analyse them to develop the most effective strategy that will address the issue globally.			
Is PSMU a significant global problem?	To identify the prevalence of social media usage and PSMU in different parts of the world	Review of the international literature	Worrying global trends of social media addiction and/or PSMU.
Is there the urgent need to address PSMU globally?	To identify the various negative effects of PSMU		Threats and negative effects of PSMU.
What are the strategies in place that are tailored towards addressing PSMU among the young population globally?	To explore the various attempts made to combat PSMU in teenagers		Technological, legal and educational means of addressing PSMU in teenagers
What is the most effective way of addressing PSMU in the young population globally?	To evaluate the strengths and weaknesses of the attempts made to come up with a framework that should be more effective than the rest.		Intrinsic Social Media Literacy (ISML)

### Search strategy

Our search strategy is in two phases. The first is the trial phase and the second is the actual search for articles. The trial search or pilot scheme search was first conducted with the keywords “combating Problematic Social Media Use” on google scholar to identify the databases which have massive collections of articles on the theme. Seven databases were prominent in bringing out articles of this theme. After this trial phase, these seven databases together with google scholar and google search engine were searched in the second phase of this strategy to get articles which are appropriate for this research. Therefore, the databases used as categorized into academic databases and the grey literature are highlighted in Table 3 below.

**Table 3.** Categories of sources of articles included for this research.

Academic databases	The grey literature
Academia.edu Researchgate Science Direct Springer Taylore & Francis Wiley Library Online	Google Search Engine Google Scholar nih.gov (National Institute of health)

### Keywords

The following keywords were used to search for articles across the nine sources already highlighted in Table 3. The keywords were (1) “addressing problematic social media use among teenagers globally”; (2) “consequences of problematic social media use”; (3) “legal means of addressing problematic social media use”; (4) “technological means of addressing problematic social media use”; (5) “educational means of addressing problematic social media use”; (6) “social media literacy” and (7) social media usage and COVID-19 pandemic “.

**Figure 3.**
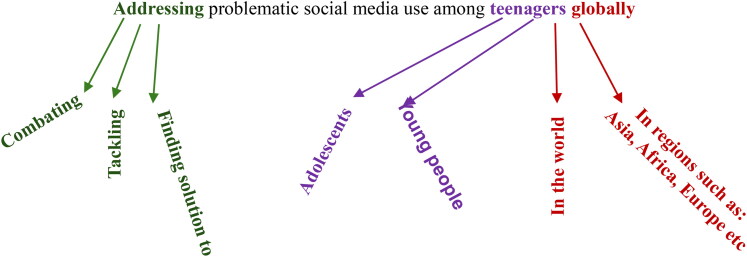
Synonyms to the Main Keywords found during Search which were selected.

The resulting articles selected based on these keyword search also had some synonymous keywords to the keywords used. Figure 3 and Table 4 highlight some of these synonyms.

**Table 4.** Synonyms to the other keywords found during search which were selected.

Keywords	Synonyms
Problematic social media use	social media addiction…… disorders of social media use… negative use of social media…. wrong use of social media…. Facebook addiction……. TikTok addiction…… Instagram addiction…… Twitter addiction……
Consequences of ….	side effects…… repercussion…. outcomes of……. result of…….
Legal means of ………………	law against…. prohibition of…. regulating…… ban to…… confiscation of phones….
Technological means of …………	the use of apps…. Applications…….
Educational means of……………….	Educators…. Teachers…… social media literacy…… awareness…. sensitization……

### Articles included

After using each of these seven keywords to search each of the nine databases, 77 articles were finally included to this research from an initial selection of 477 articles. Table 5 highlights the articles found according to the databases searched.

**Table 5.** Articles finally included after search from sources.

Databases	Articles found	Articles included
Academia.edu	61	10
Google Search Engine	124	11
Google Scholar	89	13
National Institute of Health	36	9
Researchgate	35	8
Science Direct	21	5
Springer	37	6
Taylor & Francis	43	9
Wiley Library Online	31	6
**TOTAL**	**477**	**77**

**Figure 4.**
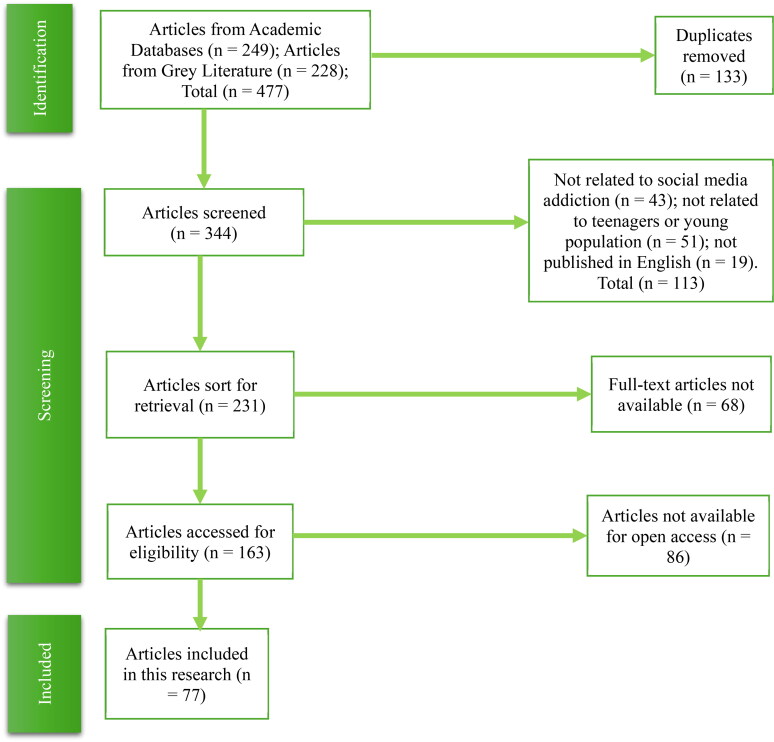
PRISMA Flow Chart of the Literature Search, Identification and Inclusion for the review.

249 articles were retrieved from academic databases while 228 were gotten from the Grey Literature. A total number of 477 articles were initially selected for this study. 133 were removed because they were duplicates, leaving 344 articles available for screening. 43 articles were not related to the main theme of this research “problematic social media use” and 51 more, though related, were not specific to young population. 19 other articles were not published in English, hence, also removed. A total of 231 articles were left after screening. 68 further articles were not available as full texted publications and so removed to leave 163 articles eligible for inclusion. Finally, 86 articles were not available for open access, leaving 77 articles that were finally included for this research. Figure 4 below highlights the PRISMA flow chart of this search strategy.

### Inclusion and exclusion criteria

Table 6 below gives a summary of the inclusion and exclusion criteria when selecting the articles searched through these two phases.

**Table 6.** Inclusion and exclusion criteria.

Inclusion	Exclusion
1. Articles that are full-texted	1. Descriptive, normative articles, responding or discussing the general concepts of the issue (eg, editorial issues, personal opinions and letter to the editor) and Book reviews
2. English language published articles only	2. non-English publications
3. Original research articles	3. Articles that are monetized
4. Systematic and scoping reviews, original research articles, meta-analysis, longitudinal studies, qualitative and quantitative studies.	4. duplicates
5. Articles or press releases from Grey literature discussing specific events to show the existence of the issue	5. Articles on bio-medical analysis/study of social media addiction
6. All of Title, Abstract, Introduction and conclusion relate with the theme of research	6. Online materials that are not available as full text (eg, published Abstracts or book chapters with intentionally missing pages).

### Outcome

In the “introduction” segment, the effectiveness of public relations strategies, not only for image or organizational reputation, but also for solving social issues have been highlighted. Also, some of the issues that result from PSMU have been highlighted. In subsequent segments of this study such as the “discussion” section, the strategy of social media literacy, both in the classroom and as technologically incorporated will be discussed. Therefore, a new strategy of social medial literacy is to develop messages that are tailored towards enlightening potential “problematic social media users” by creating persuasive, informative, facilitative or cooperative problem-solving information tailored towards warning against the negative effects of PSMU as defined in the “introduction” section of this study. This new strategy should also makeup for the weaknesses of the other attempts that already address PSMU which would be discussed later. This expected outcome is what will result to the Intrinsic Social Media Literacy, ISML.

### Limitation

**Figure 5.**
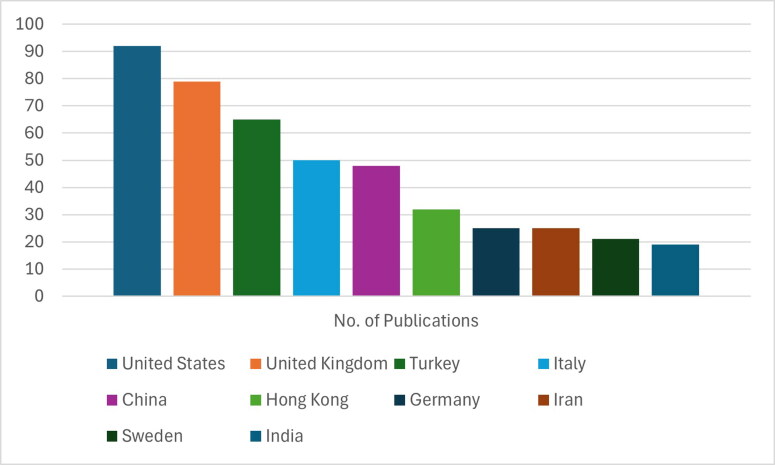
Global Research dispersion of social networking sites in relation to PSMU. Source: (Pellegrino et al., 2022, p. 6).

The only limitation of this research is that the literature included heavily focus on high-income countries. PSMU in regions such as Africa and Central and South America, Australia, New Zealand and a huge part of Asia are either not researched at all or not satisfactory researched. A lot of the studies on Asia that were found were not accessible, not full texted or not in English. Figure 5 below gives an idea of this trend indicating the need for more quantitative and qualitative studies in these regions in order to have a good global coverage of any intervention recommended.

## Discussion

It is paramount to note that if PSMU or specifically, social media addiction becomes a diagnosis in a young patient, the only option is to seek professional help from a therapist or counsellor who specializes in addiction treatment (Esmaeili Rad & Ahmadi, 2018). The common treatment in this case is the cognitive-behavioural therapy (CBT) which helps improve the young patient’s thinking and behaviour (Brand et al., 2022, p. 3-4). Therefore, in this study, the aim is not to replace this treatment strategy but to focus on a “preventive angle” of PSMU. As it is popularly said, “prevention is better than cure”, hence, finding the most effective preventive strategy whilst justifying it, is what this review is about. Therefore, the options to be critically analysed will be without the psycho-clinical or bio-medical management strategy of PSMU as the other options such as the legal, technological and educational as illustrated in the conceptual framework would be analysed. There are strategies that are combinations of two or even all the options which will also be discussed. For instance, Mass Media is a combination of the educational and technological strategies, while public health approaches are a combination of all three. By analysing their strengths and weaknesses, the critical analysis of the most effective strategy will be achieved.

### The legal strategy

This is the strategy that involves the partial or complete ban of social media use and access by teenagers and Adolescents. For instance, policy makers in the United States are being pressured by parents, pundits, media houses and organisations to put a complete ban on social media (Barkley, 2024). Also, in response to the public health emergency in tackling the tech- and PSMU of French teenagers, France drafted a legislation in 2017 that will enforce the limitation of its children under the age of 16 to access mobile phones and social networks. They will also need their parents’ approval to open accounts in any social network and their parents strict monitoring while using these accounts. This legislation comes from the backdrop of the already existing prohibition of the use of mobile phones in French classrooms as more prohibition has included breaks, mealtimes and between lessons. Interestingly, the tech community in France was in full support of this ban against the warnings from experts on the practical difficulties of controlling children’s internet and social media usage (Agnew, 2017).

In November 2024, the Australian government enacted a ban on under-16-year-olds from using social media, alongside slapping tech companies with fines up to $32.5million if they do not comply. Though YouTube, Gaming and messaging platforms are exempted, the ban will include Facebook, Instagram, TikTok, Snap and X (Ritchie, 2024). As the world is carefully watching this ban as it unfolds, recent history shows that a similar legislation in Utah, USA was enacted but overturned by a federal judge on account of being unconstitutional. Furthermore, the law is not clear and quite complex, hence, the demand for more details from the active proponents of the ban- people such as the prime minister, Anthony Albanese and Australia’s communications minister, Michelle Rowland need to enlighten the public more on specifics in relation to the ban (Ritchie, 2024). There are many more reasons why social media is partially or completely banned in some countries and Table 7 below shows some of these reasons.

**Table 7.** Complete and incomplete ban to social media around the world.

Social media companies	Countries involved in the ban and reasons
***Complete Ban***	
Facebook	Bangladesh, China, Cuba, Egypt, Iran, Mauritius, North Korea, Pakistan and Vietnam. The reason is mainly politically motivated to avoid the people’s access to organised political rallies. Other reasons include the pronunciation of Facebook in Mandarin: “fei si bu ke”, which means “you shall die”. In North Korea, the ban is to block the flow of information both domestically and internationally.
WhatsApp	Brazil and India which also banned Facebook alongside WhatsApp.
Viber	Bangladesh: to stop online criminal activities.
YouTube, Flickr, Wikipedia and Twitter	Pakistan: due to national security and the promotion of blasphemous materials on these sites.
***Partial Ban***	
Blogger, Twitter, LinkedIn, Viber, Skype	No access during working hours in some provinces in China such as Beijing, Shenzhen, Inner Mongolia, Heilongjiang and Yunnan Province.

Source: (Tuncay, 2018).

What have been the outcomes of these bans? In France, despite the enactment of the law in 2017, by 2023, the French National Commission for Technology and Freedoms stated that the law had been ineffective in limiting French kids’ access to social media. As a matter of fact, the situation had even worsened as users as young as eight years old are opening accounts in social media sites and more than 50% of children aged 10-14 use social media platforms like snapchat and Instagram (Scottish Legal News, 2023). In the Asian countries that have enacted and implemented these bans on social media, a lot of their young English-speaking citizens still have Facebook accounts and are actively using it in these same countries. This is made possible using Virtual Private Network, popularly known as VPN. VPN connects one’s internet-networked device to a remote server, creating a point-to-point tunnel that encrypts one’s personal data and masks their IP address so that they could sidestep website blocks and firewalls on the internet (David, 2015; Stewart, 2013; Tuncay, 2018). Just immediately after banning WhatsApp in Brazil, the people took to the streets in heavy protests which led to the immediate reversal of the ban (Murgia, 2015).

Therefore, social media ban is not effective in reducing its excessive usage by teenagers or adolescents. This means that it will also not be effective in addressing PSMU as well. If justifications as serious as fighting child sexual abuse, infringement of privacy, fighting online crimes, and other reasons of political nature that will attract significant degree of attention and resources from the government could not stop a population’s access to social media, PSMU will hardly be different. Some studies done have suggested the ineffectiveness of banning social media. For instance, banning social media in secondary schools have no effect on the academic performances of the students. However, well-designed instructional materials for developing digital competence are needed to engage students and enhance their academic performance in the online environment, since, in a place like Cyprus, social media ban created negative psychological effects (Li and Ranieri, 2010). This recommendation is also known as social media literacy. Vulnerable teens like the LGBTQ-teens who might be isolated, mocked and insulted with gay or transphobic slurs or a coloured-skin teen isolated in a neighbourhood dominated by a different race, experiencing racism and regular microaggressions may turn to social media as a haven where they interact with others like them. Social media may provide the needed community that understands the stressors they go through on a regular basis. Therefore, severe restriction or an outright ban will take away this protective feature, thus stripping away that needed sense of safety which could lead to outcomes of suicide. Digital literacy from an early age which involves teaching children the right way to use social media whilst learning how to interpret technicalities around its usage and growing to be critical thinkers of their media use is the only solution in effectively addressing this issue (Coyne, 2023; Nevard et al., 2021). Again, this strategy, in other words is social media literacy.

Does this mean that the law or legislation does not play a role in addressing PSMU? Long before the year 2024, there has always been a public outcry especially in the USA for governments to regulate social media just as it was the case with alcohol and cigarette smoking. In the United Kingdom, children are more targeted to be protected from social media than any other demographic. This call was a product of the concern raised on account of the growing number of young problematic social media users in the USA and the United Kingdom. Despite having the US’ children’s Online privacy Protection Act which carries an age limit of 13 in all social media sites, an increasing number of Under-13s were not only found to be active social media users, but also found to be problematic users. This shows an ineffective enforcement of such a policy. Therefore, lessons were adopted from the “18-plus pornographic websites” which included the proof of age and identity before subscription, and this has since been applicable in social media platforms (Lundahl, 2021, p. 1109). From the EU-based Alliance to better protect minors online to Australia’s eSafety Commissioner and to the United States’ Children’s Online Privacy Protection Act, various countries have taken measures in a policy level to address social media threats and risks. Whilst some are self-regulatory initiatives, others are more of a legislative approach. The aim of all these bodies is to regulate harmful contents such as violence or sexually exploitative contents and conducts such as cyberbullying and sexual extortion. Unfortunately, what seems to be missing in all these policies is PSMU or social media addiction itself (Alexander, 2022; Meola, 2020). The United States launched the Social Media Addiction Reduction Technology (SMART) Act targeting social media companies in their recommended interventions. But would this legislation gain support from other legislators, the media and the public? Until this happens, the public policy approach on social media companies remains largely normative and difficult to implement (Lundahl, 2021, p. 1105). Similar situation is the Australian ban on social media use by under-16s, which has been considered one of the world’s strictest laws. Critics warned that it is an instrument that is too blunt which may push the children affected to “less regulated corners” of the internet. Moreso that those who flout the rules to use social media will not be punished. Among the critics are representatives of internet giants such as Google and Snap who believe that the ban will not be effective in making kids safer. And then, there are aspects of the bill that may violate human rights as pointed by X’s representatives, when questioning the bill’s lawfulness (Ritchie, 2024).

### The educational strategy

From the previous strategy, recommendations of digital or social media literacy and education have already been given, showing the huge importance of educating the young population on social media. The educational strategy consists of the classroom education and the community awareness campaign. Another terminology used for this is “social media literacy” (Gammon & White, 2011). In response to the high rate of tech- and PSMU among French teenagers, Rachel Delacour, an entrepreneur and the then co-president of the industry body- France Digital, stated that the solution to the problem is not regulation but better education. The children’s critical spirit needs to be promoted and their ability to analyse and distance themselves from the excessive usage of their phones need to be taught, encouraged and instilled in them (Agnew, 2017). School and community-based programs aimed at limiting social media usage have been proven to be effective in improving behaviours associated with PSMU as well (Strieter et al., 2019). For instance, Ofcom, together with the National Literacy Trust, the Guardian Foundation, the Personal, social health and economic education Association (PHSE), and funded by Google, launched an initiative called NewsWise in September 2018 in the United Kingdom. This initiative is a free, cross-curricular news literacy project for years 9 to 11 across the United Kingdom, providing teachers with curriculum-based lesson plans, online resources, school workshops and platforms for interactions with professional journalists. The BBC also launched a similar program in the same year to support young people identify real news content, giving all schools access to online materials and the iReporter game (Science & Technology Committee, 2019). The education strategy enhances the “competence-performance” output of Adolescents in relation to having improved social media behaviour (Festl, 2021, p. 252). Enhancing competence also enhances knowledge and skill which in turn encourages critical thinking because knowledge and skill are needed to identify, evaluate, analyse and produce media messages. This is also consistent with earlier recommendations, especially the one made by Rachel Delacour. Social Media Literacy through education enables the user to adequately identify and understand risks or threats and hence avoid negative experiences online (Festl, 2021, p. 252-3). On the other hand, public awareness campaigns provide the necessary education on the impact of the excessive use of digital media whilst promoting healthy behaviours in this regard (Marchant et al., 2017). Also, the contributions of social media giants in designing and promoting behavioural reinforcement and problematic use, specifically on platforms commonly used by the youth should be discussed, deliberated upon and even debated openly and in public discourses (Abi-Jaoude et al., 2020) so that clear messages and awareness, using the informative, persuasive and cooperative problem-solving strategies, as discussed in the introduction, to address PSMU may be accomplished.

But the educational strategy also has its weaknesses. One of such is its suitability for the developed or high-income countries. The educational strategy in class will require human, technological and infrastructural resources to develop and maintain, since it is not only communicative in nature but also participatory too. The most attainable attempt to reach global coverage under this strategy is the community awareness campaign. This too will require a long-term implementation which will need resources, coordination, consistency and maintained level of passion (Hinde et al., 2015; Seymour, 2018). If not with the help of foreign non-governmental organizations, implementing such campaign on an issue as “PSMU” which may not be taken too seriously in parts of the world such as Africa, will most likely end up unsuccessful. The use of “neutral language” also is essential for this strategy to be effective. In the Science and Technology Committee of the UK’s Parliament’s (2019) report, heavy emphasis was laid on being all-inclusive and non-judgemental when recommending community approaches in tackling PSMU. This may be difficult to achieve in most societies, especially those that are heavily influenced culturally. Also, these educative strategies will need a lot of collaborations and joint efforts, coordinating these collaborations to achieve effectiveness may be an issue, even for a high-income country, just as it is the case in the United Kingdom’s community-based approaches in handling PSMU (Science & Technology Committee, 2019). The education strategy be it in classroom or as a community campaign instrument is a highly technical one and one area of necessary technicality is research. For instance, Ofcom is responsible for the promotion and implementation of research on media literacy under the Communications Act 2003 in the United Kingdom (Science & Technology Committee, 2019). And research is a huge challenge in most low- and middle-income countries of the world (Barrios & Mano, 2021; Steinert et al., 2021). Therefore, the education strategy is effective regionally, especially in Europe and North America, but globally, it will still fall short.

Classroom social media literacy will not work solely on the educative strategy unless other strategies are implemented along with it. This means, having many components of the strategy to implement and monitor and hence reducing the chances of effectiveness. For instance, the family remains the primary and, most often than not, the first place of children’s contact, access and experiences with social media. Therefore, parent’s educational and mediation strategies are very important in influencing a child’s social media use and hence, must be included to support classroom social media literacy. Parental mediation is therefore, any mechanism used to control, supervise, or interpret content in relation to social media and these mediation strategies are: (1) restrictive mediation which is the setting of rules and regulations on the child regarding time and contents, (2) co-viewing/co-using mediation or active mediation which is jointly accessing, evaluating and consuming social media contents by both the parents and the child, and (3) technological mediation which is the use of monitoring software (Festl, 2021, p. 254-55). A parent who is not cooperative enough, interested enough, knowledgeable enough or intentional enough is already setting the path to failure for this strategy (Festl, 2021, p. 255).

### The technology strategy

The use of technology online is a strategy of addressing PSMU. There are some technological tools, mostly applications that can help users to manage their social media usage. These applications must be downloaded on the device running the social media applications. Secondly, the application must be installed and activated for it to work. Table 8 below shows some of these Applications and their functions.

**Table 8.** The most commonly used social media monitoring applications.

Applications	Use
App Detox	This App allows users to set rules for their App usage, such as limiting the number of times an App can be opened in a day or setting a time limit for App usage.
Flipid	This App allows users to lock themselves out of social media Apps for a set period of time. Users can also create custom challenges to help them stay off social media.
Freedom	This App blocks access to social media Apps and Websites for a set period of time. Users can also create custom blocklists while using it.
Moment	This App tracks the amount of time users spend on their devices and provides daily reports. Users can also set daily limits on their device usage.
Screen Time	This App allows users to set limits on the amount of time spent on social media Apps. Users can also schedule “downtime” during which only certain Apps are available
All Applications are available on Android devices and all except App Detox are available on iOS devices.	

Information retrieved from their Google Play Store pages and Associated reviews of each Application.

This strategy is an excellent one that monitors a teenager so that the excessive use of social media can be curbed. However, it is not effective because, one needs to download the Application on their phone before it can be used. Even if downloaded and installed, one will need to activate the App after installation. Therefore, there is the possibility of the teenager to always deactivate the App whenever they want to use social media. Even if this is not possible, a very smart kid would not mind always deleting and re-installing the App before and after social media use. But even if successful in monitoring a child, the restrictive use of social media monitoring technology will massively reduce the risk of exposure to online threats but also will reduce important online opportunities to the child such as more diversified sources for schoolwork and entertainment (Glüer & Lohaus, 2018; Livingstone et al., 2017). This will most likely extend to negatively influencing their internet usage skills in informational, social, critical and technical issues (Festl, 2021, p. 254-55). This is an unfair and unwarranted punishment on the child.

### Combinations of the strategies

#### Mass media

Mass media is education and technology strategies combined. Mass media is so powerful to the extent it can influence structural changes in a society. Mass media is of great influence in improving the behaviours of the young population in terms of PSMU. However, Mass media could instigate moral panics, manipulating governing bodies to implement excessive regulations to contain the threats seemingly posed by addictive individuals. Therefore, mass media could establish an issue to the consciousness of the public, legitimizing the perspective they push, but in the process, setting an Agenda. The media is highly influential in both driving public policy and encouraging attitudes for, or against addictive behaviours. Also, by creating moral panics, the media generates the framing of addiction, and this may end up being counter-productive (Lundahl, 2021, p. 1103-05). Then, the sensationalism and exaggerations that come with mass media reporting which further fuel stigmatization and discrimination against problematic social media users is another huge challenge associated with this strategy. As from 2017, mass media came out even stronger, louder and more aggressive on PSMU. But this time, they blame social media companies for intentionally adding addictive components to social media (Lundahl, 2021, p. 1107). The “reward mechanism” of liking posts, comments on Facebook, in particular, is an opportunistic invention to the vulnerability in human psychology. The “liking” or “reaction” to a comment is an insects-mammal-like dopamine process that encourages the behaviour of “commenting” and “posting” to continue. In this way, more of the consumer’s time and conscious attention are consumed. This mechanism proved effective as Facebook users became obsessed with the number of likes their social media activities get. Other articles implied how the insiders themselves are aware of these manipulative innovations and inventions in social media as Steve Jobs and Bill Gates, the CEOs of Apple (late and former) and Microscoft respectively restricted their own children from social media usage. Whilst it was a welcomed development for the mass media to share this information, the comparisons of these CEOs to the Tobacco Industry, gambling and drug barons gave the whole issue a new look that is more damaging and audaciously inciting towards the discrimination of social media problematic users. The outrage even became worse when Facebook for Kids was launched as this meant that these “terrible CEOs” are now targeting children of younger ages to be victims of addiction (Lundahl, 2021, p. 1108). Therefore, the use of mass media has the tendency to use “bias language” as discussed in the introduction of this study. The use of bias language may create moral panic and the sensationalism of the issue. This then means that mass media as a strategy has a huge weakness in addressing PSMU.

#### Public health strategy

Community awareness campaigns which are similar in operational delivery to Public Health promotional strategies have previously been discussed. But more sophistication and more multidisciplinary approaches are required for any community awareness programme to be fully characterized as a Public Health strategy. A public Health strategy mostly employs health promotional techniques in solving addiction in general. These techniques require all the techniques of public relations: informative, facilitative, persuasive, cooperative problem-solving and power strategies; and all social media preventive strategies: legal, education and technology to implement. Health promotional techniques use models, for instance, the McElroy Ecological Model (Ainsworth & Der Ananian, 2020), or the Wheel of Behaviour Change COM-B Framework (Michie et al., 2014). These models require empirical and highly professional methodology. Applying these models will require highly skilled professional collaborations, multi-disciplinary approaches, financial and technological investments, long-term planning, implementation, monitoring and evaluation, consistency in communication, policy making and community engagements. For a social issue like PSMU, low- and middle-income countries will most likely not give it the needed attention and investments via this approach. Also, Pagoto et al. (2019, p. 6) mentioned the challenges to public health approaches for addressing PSMU which are, (1) the negative sentiment surrounding social media and the villainization of social media giants among the public and scientific communities; (2) under-developed research environment; (3) poor social media data access and (4) the cohesive academic field which is lacking. These challenges are making it difficult for the public health approach to be effective.

### Social media literacy

#### Project COURAGE

The project Courage is another technology and education combination of strategies that can be used to address PSMU. COURAGE uses artificial intelligence to create awareness against social media threats and addiction. As a virtual learning companion, it could identify and label toxic contents, fake news and regulate information overload, all to create the necessary awareness to a learner or new customer using it. By using the informative, facilitative and to some degree, the cooperative problem-solving strategies of public relations, this model communicatively engages the young user and instils in their minds the ability to analyse and distance themselves from the excessive usage of social media whilst encouraging a critical spirit (Donabauer et al., 2022) which is, yet again, the recommendation of Rachel Delacour to the French Authorities on tackling the social media and tech addiction of their children. The COURAGE project has a virtual Learning Companion which operates on a Natural Language processing framework (NLP) which effectively communicates with its learner in an adaptive feedback mechanism. These frameworks are highly advanced tools that make predictions and analyse textual and image contents which can identify sentiments, emotions, hate speech, fake news, irony and sexism. Also, by using image algorithms, they can predict body mass index (BMI) of individuals, which is helpful in identifying threats from beauty stereotypes. They also have advanced algorithms for gender identification and object detection (Donabauer et al. 2023, p. 401-02).

#### BBC iReporter

A similar application of this strategy is the BBC iReporter Game. This is a platform for young people which makes them act like real time journalists. The Artificial Intelligence Application is an interactive Game that has a virtual interface which will require a smartphone, tablet or desktop to play on. The first Screen on the interface reads “*Your role as a BBC Journalist is to cover a breaking news story- publishing your story to a BBC Live site. Your story will be judged on how well you balance accuracy, impact and speed.”* Clicking on the “next” buttons places the young person in more engaging positions to play the game. All these will occur with the typical BBC News background music. The learning outcomes are: (1) young people will question which news sources are trusted sources and how to carryout basic checks and (2) they will have a better understanding of the Pros and Cons of using Social Media sources as individuals, professional journalists and as well as news organisations (BBC iReporter 2022n.d). This strategy is highly skilled which needs huge investment in human capital, finances and technology. That is why it is in use in regions like Germany, the United Kingdom, Italy and Spain. As a global challenge, achieving effectiveness in solving PSMU through this strategy will not be attainable in every part of the world. The question now is, how can a strategy that will disseminate social media literacy which is cheap, easy to implement and having an expansive reach to every problematic user or potential problematic user globally be developed? This is where “Intrinsic Social Media Literacy,” ISML, comes in.

### Intrinsic social media literacy (ISML)

Developing the ISML strategy does not only aim at improving on social media literacy strategy but also to address the weaknesses of the other strategies analysed in order to boost the effectiveness of intervention on a global scale. Therefore, Table 9 below summarizes the weaknesses of the other strategies as discussed.

**Table 9.** The weaknesses of ongoing strategies tailored towards addressing PSMU.

Strategies	Weaknesses
The Legal strategy	Incomplete or partial bans will only make users beat the system via the use of VPN to continue using social media.
	Bans may infringe on human rights and hence be unlawful to some degree.
	Bans on teenagers may instigate more audacity for more users of even younger ages to become social media users just as with the case of France.
Education	Mostly suited for high-resource countries as it is too expensive and highly technical to implement.
	The classroom component of this strategy may not be effective if the parents of the teenagers involved are not on board with it.
Technology	This plays on the teenage users’ hands as they can install, uninstall, activate and deactivate the App whenever they want to before and after social media use.
	If successfully implemented, it will drastically limit the teenagers’ positive social media experience.
Mass media	This will instigate moral panics, sensationalism and exaggerations that will heavily influence the discrimination against problematic social media users.
Public Health Strategy	Mostly suited for high-resource countries as it is too expensive and highly technical to implement.
	The villanization of social media by scholars, poor research on social media dynamics and experiences, inadequate data access and the lack of cohesion in the academic field already makes this strategy a difficult one to implement to effectiveness.
Social Media Literacy	Mostly suited for high-resource countries as it is too expensive and highly technical to implement.

**Figure 6.**
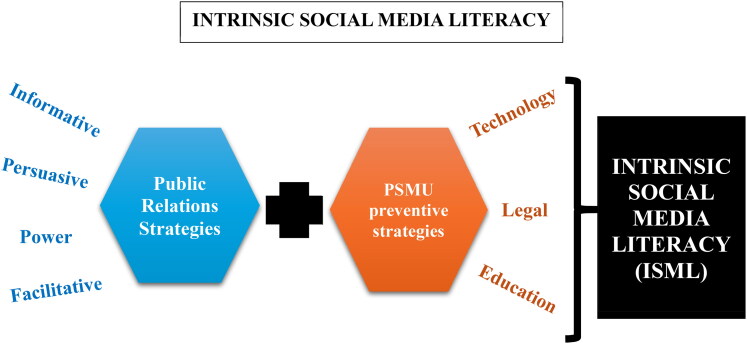
Intrinsic Social Media Literacy Model to Address PSMU.

ISML is recommended as a novel approach as of the time of writing this review. The word “Intrinsic” means to belong naturally, essential or relevant to the source that a thing is coming from (Oxford Dictionary, 2000). ISML is social media literacy that uses visual advert messaging from the same social media applications to educate on social media and warn against its problematic use. From the introduction, side effects of social media are Salience, mood modification, withdrawal, relapse and conflict. Threats are filter bubbles, echo chambers, digital wildfires, impulsivity, confirmation bias, social reinforcement, backfire effect, emotional load, anonymity and Deindividuation. Specific threats of content-based nature are toxic contents, fake news or disinformation, bullying, hate speech, stalking, discrimination, radicalization, sexism/sexual harassment, objectification and beauty stereotypes. Finding a way to incorporate the knowledge of these side effects and threats associated with social media use will go a long way in addressing its problematic use. It is possible to bring about Social Media Literacy by adopting public relations strategy of passing information or messaging from the Social Media Applications themselves and this model is what we call, Intrinsic Social Media Literacy. Figure 6 above shows the model that will be implemented by doing so. The use of technology is the social media application and platform itself that will be used. Therefore, for every fifteen minutes that a social media platform, let’s say Instagram, functions, a compulsory thirty seconds “social medial literacy” advert that cannot be skipped should be played. Such type of compulsory advertising is already practised by Facebook and YouTube (Joa et al., 2018; Paid Media Pros, 2023). The Legal approach is needed so that governments will enforce the implementation of this literacy tool if the social media companies want their products to be used in their (the governments) territories. The educational aspect of this approach is the social media literacy itself. This literacy could be direct or indirect. The direct literacy is the messaging that defines or tells a story about the side effect or threat of PSMU, using this definition to warn the user that is watching. In the direct style, the threat or side effect is mentioned when defining or telling a story about it. In the indirect messaging style, the side effect or threat is never mentioned and hence no definition or storytelling. This style is a straight-to-the-point warning about the features of the side effects or threats in question. While the direct style messaging will use the informative and power strategy, the indirect style may use any of, or a combination of the informative, persuasive, power and facilitative strategies of public relations to pass the message of social media literacy across to the user. Table 10 below summarizes the characteristics of these two styles of ISML messaging.

**Table 10.** Messaging styles in intrinsic social media literacy.

Direct	Indirect
Messaging involves the definition and storytelling of the side effect or threats of PSMU.	Messaging is a direct warning.
The threat or side effect of PSMU in question must be mentioned.	The threat or side effect of PSMU must not be mentioned.
Messaging uses the informative and power strategies of public relations only.	Messaging may use any of all the strategies of public relations.

A young vibrant person of the age bracket 13 to 19 should be the advertising model in these adverts and this person in the short 30-seconds clip should, in a wonderful countenance, relay the message. Here are some messaging examples and how they intend to solve some social media threats whilst demonstrating social media literacy.

#### Examples of direct messaging

1. “Are you using social media as your comfort zone to reduce stress, anxiety, helplessness, hopelessness, restlessness or even depression? If yes, you are using social media in a problematic way. The experts call this “mood modification” which is bad for you. Talk to someone, reconnect with loved ones- friends or family around you and reduce your social media presence. This may not end well for you if you continue this way. So, you have to follow my advice and help yourself.”
2. “If you are not on social media, do you feel stressed, restless, troubled or irritable? NAAAH! This is not good at all if your answer to that question is yes. You are gradually becoming a problematic user of social media because what you are experiencing is called “withdrawal”. You need to step back, re-evaluate your social media usage and ensure that you cut it down drastically. Then connect more with your loved ones, engage more in your hobbies, have outdoor fun, travel, take holidays, mingle and be happy. Life is beautiful, but social media can take all that beauty away from you, so, please don’t let it.”
3. “Hey you! Yes you! Yes, you that is looking at me, I am talking to you. Have you heard of the term “filter bubbles”? Aha! I knew it. Filter bubbles are algorithms created and used by social media Apps to keep sending similar contents to the ones you have been watching, to your feeds and notifications tab. In this way, you suffer from the lack of diversity of information, perspectives and opinions. Have you noticed this as a trend on the contents recommended for you to watch? If yes, trust me when I tell you, this is dangerous for you. You could easily be manipulated or brainwashed. Be intentional about searching for broad themes on issues. Never take one side of any social issue or trending topic hook, line and sinker. And make sure that you are aware of platforms that propagate certain agendas, especially controversial ones. A brainwashed kid today can be an Adult extremist tomorrow. Diversify your contents, follow all sides of an issue and reduce your social media presence to free yourself from easy manipulation from vicious people and groups.”
4. “I have a friend who is so engrossed in a certain ideology. He sees himself and his fellow adherents as pure, the only true stewards of humanity and their ideology as the purest form of thinking and living on earth. As noble as this was, my friend got intoxicated into this, thanks to social media where he connected with other followers of his ideology. He then became intolerant, unaccepting of other views, unnecessarily aggressive, and a strong hater of contrasting opinions. Social media amplified his bigotry and tribal perspective and so, he antagonizes any form of online discourse that allows for diversity. The experts call this problem “Echo Chamber.” We are in a world of so many contrasting opinions, perspectives, ideologies and beliefs. All these ideologies may mean well for humanity but if my friend’s circumstance is what you are also going through, then, you will need to take a step back from social media activities, look for a respected person around your neighbourhood or circle to talk to and then reorient yourself. Life is nothing without diversity. Education, growth and maturity comes with embracing diversity and this also includes opinions whether you believe them or not. Learn to put your legs in the other person’s shows even if you won’t use them to walk and understand the dynamics of other ideologies or contrasting perspectives. This will make our society healthier, more accommodating, progressive and peaceful. Lastly, you may come back to social media, but you must drastically reduce the time you spend on it.”

#### Examples of indirect messaging

##### Messaging that targets digital wildfires

“Nine rumours in one day! Damn! What is social media turning into? Have you experienced it too? Or you don’t ever know how to identify fake news and rumours? You need to be careful with what you read online. But most importantly, reduce your online presence on social media so that you won’t be in trouble from what you read, watch or listen to. I care about you, and I am sure your loved ones do as well. Please, reduce your social media usage and find yourself far from trouble.” This messaging uses the informative and persuasion strategies of public relations.

##### Messaging that targets impulsivity

“Hey you! Do you love social media just like I do? Do you think you love it more than you should? You may not be able to answer my questions, but I will recommend you ask at least the three closest persons to you- they may be your family, or your friends, please ask them. If they think you cannot do without social media, maybe it’s time for you to cut down on your social media presence and work on yourself. If these two are difficult to attain, then maybe you need to seek for professional help. I do not want you to suffer similar conditions like those in addiction, or do you want to?” This messaging uses the informative, facilitative and power strategies of public relations.

##### Messaging that targets salience

“Hello lovely. When last were you away from social media? Were you eager to return to it when you left? Do you know that it may be a sign of already becoming a problematic user? You do not want to be a problematic social media user, trust me on this because it will affect you, your relationships and your freedom. Just make sure you make healthy use of this space okay!” This messaging uses the informative and persuasive strategies of public relations.

##### Messaging that targets conflict and withdrawal

“Aha! Isn’t social media so much fun? I hope you didn’t rush back here after leaving. Listen to your loved ones if they are stopping you from spending so much time here, or else that restlessness/irritation/troubled mind of yours when not here may lead you to even worse states. I know you do not want that. I would not want that for you either. Please dear, reduce your stay here, mingle more with loved ones, find a hobby, build yourself and see how happy and satisfied you will be. I want all of us to be happy, so I will give you the next two minutes to press the “sign out” button, leave now and go be with your loved ones.” This messaging uses the informative, facilitative and power strategies of public relations.

##### Messaging targeting filter bubbles, echo chambers, backfire effects and emotional load

“Hey dear! You look cute today [smiles]. Just this week alone, I have faced attempts from information sources trying to put me in a box, can you imagine that! Democrat versus Republicans, Right wing versus left wing, liberal versus conservative, religious versus free thinkers, capitalism versus socialism, rap versus pop, comedy versus thriller, man versus woman, boy versus girl…. I am tired already…. Gosh! If it were you, won’t you be? I have had an open mind for the past few months, and I have come to appreciate all views. I have also cut down on my social media presence and guess what! I do not get these views being chocked down my throat anymore. You can do the same love. Be open-minded and reduce your social media presence and see yourself less exposed to these manipulative, bigoted and radicalizing posts and news.” This messaging uses the informative and power strategies of public relations.

##### Messaging that targets mood modification

“Hey lovely! I did not know I will bump into you today. I was once too comfortable on this same platform because I thought it kills my anxiety and restlessness. Did you know that this later affected the sweet relationship I had with my mum? Maybe you should cut down on the time you spend here too so that it does not happen to you. If you cannot, then please darlin, seek professional help. Throwing kisses and love to you darlin.” This messaging uses the informative and persuasive strategies of public relations.

##### Messaging that targets relapse and conflict

“How many times have you come on this platform just today? [laugh in a cute way] This was me three months ago. You know what? I had to cut down on my social media presence to two hours a day, from eight hours, could you imagine this? Now, I spend more time with the people that are important in my life, I paint more because I love painting and social media never allowed me to adequately do what I love. It will amaze you that I even had time to visit Machu Pichu! I know you are wondering what this is…. It is one of the wonders of the world, the best-known archaeological site in all South America, situated in Peru. I plan on visiting the Northern lights in Iceland soon [cute laugh again]. Don’t you want to be like me? Be free and have the time to do what you love doing? I think you can, but if you stay glued to social media, you won’t be able to. I guess it’s time to cut down.” This messaging uses the informative and persuasive strategies of public relations.

##### Messaging that targets content-based threats

“Hello dear…. Hmmm! See how engrossed you are on social media. I hope you are not falling for friends’ requests from unknown persons, or strangers asking for your personal information, asking you to privately chat with them, to see you through the webcam or even wanting to meet with you. Have you come across links you are not sure of? Or perhaps you copy and paste information you aren’t sure of. Please my dear, avoid these circumstances. You’ll do yourself much good if you even reduce your social media presence so that you will less likely fall victim of these cyber threats.” This is the informative strategy of Public Relations.

ISML may not be necessarily left in the hands of nations to execute. The World Health Organization may solely adopt this approach, create the messaging system, hire the young, vibrant, good looking and eloquent teenage models and create the adverts themselves. Then nations can simply just key into the intervention, thereby mandating these messages to be compulsory in any territory that partners with the World Health Organization on account of this intervention. But if it may be left to the discretion of nations, these messaging should be developed by the health authority or/and orientation/communications ministries responsible in the country of concern to develop the adverts and implement them. There should be as many of these messages as possible, allowing different young, happy and receptible models to feature in the advert and then sent to the social media companies operating in the territories to run them frequently (one advert per 10 minutes of continuous social media use, for example).

#### Benefits

The benefits of this model are, (1) this model will reach its target population and much more, provided there is access to social media, irrespective of how remote that part of the world is. Therefore, problematic, and potential problematic users, and all social media users around the world will benefit from this literacy model. (2) ISML is cheap, easy to implement and easy to monitor and evaluate unlike in the legal and public health strategies. (3) ISML is not restricted in time and space just as in the educational and technological strategies, hence functions always. (4) ISML does not give room for manipulation, exaggeration, sensationalism, moral panic or any form of Agenda. The health Authority in charge of the region in question will take charge of the messaging to ensure that none of these happens whilst giving the social media companies no chance to propagate any Agenda that will give them undue and “unholy” advantage to the detriment of young users. This regulation will even be better if under the control of the World Health Organization. (5) ISML does not hinder other existing or intending strategies of addressing PSMU from implementation. This model can go on alongside any interventions in place in any country or region. (6) The social media young user has no power or control over this model, unlike in the technological strategy, since this model functions as a compulsory advert that cannot be skipped. And because the advert is short and interesting, the young user can wait, watch and listen to the messages. Several of these messages every day, every week, every month and every year of social media usage will create an impact in the young user’s life, be them problematic users or not.

## Conclusion

This study is aimed at exploring the international literature to identify problematic social media use, PSMU, as the major global problem in teenagers and using public relations strategies in addressing it. The negative effects and threats that social media addicts are more exposed to than usual users were briefly discussed. Since the issue being dealt with is an addiction-like situation, medicine and in particular, psychiatry will most likely always be the first line of thought when thinking of a solution to this global menace. But biomedical means, though cannot be replaced, are not the only approaches in addressing PSMU. PSMU can also be addressed through legal, educational and technological means, especially preventively. Public relations also have a huge role to play in addressing this issue. The strategies that combine some or all these approaches such as the Mass Media strategy and Public Health strategy were also discussed by critically analysing their strengths and weaknesses to have a clear understanding of how effective or ineffective they have been in tackling PSMU. The partial or complete ban of social media has been proven to be ineffective, as legislation on the control and regulation of social media remains highly normative and difficult to implement. The use of technology such as applications are a good strategy but also play in the hands of the problematic user as they will need to be downloaded and then activated to be able to monitor the young user. These manoeuvres render this strategy ineffective because the young user can decide to always deactivate or even uninstall the App anytime they want to use social media.

The educational approach requires technical skills, investments and long-term planning. Social Media Literacy that combines both the educational and technology approach is also sophisticated and will require high-skilled training of teachers to adopt. This approach is more suitable for the developed countries and hence, achieve poor global coverage. The public health approach is even a more challenging approach with similar issues with the educational approach, as a multidisciplinary setup that involves different professionals, long-term monitoring and evaluation, consistent research and sometimes policy and cultural involvements is required. This can be too expensive, highly technical and beyond the reach of most low- and middle-income countries. Mass Media can be an excellent approach, but it is prone to cause moral panics. Its liability to sensationalize, exaggerate and use “non-neutral” language can breed the stigmatization and discrimination of problematic social media users. Therefore, Social Media Literacy is the way to go, but still suffers in global coverage as it is more compatible with rich nations. Enhancing social media literacy then means improving on or maintaining its strengths whilst solving the weaknesses of the other options. This enhanced form of social medial literacy is the Intrinsic Social Media Literacy, ISML. The ISML is a simple model of social media literacy that incorporates the strategies of public relations and that of addressing PSMU to improve in the coverage and effectiveness of social media literacy, whilst solving the weaknesses of the other attempts being made to solve PSMU. This model does not stand alone as it will still need to be implemented through its legalization via policy making. Also, other interventions may continue, despite the huge potential of its effectiveness.

## Data Availability

Data sharing is not applicable to this article as no new data were created or analyzed in this study.

## Other declarations

This review has not been registered because it is only a simple review and not a systematic one or meta-analysis and hence, a protocol not prepared also.

## References

[CIT57963829] Abi-Jaoude, E., Naylor, K. T., & Pignatiello, A. (2020). Smartphones, social media use and youth mental health. *CMAJ : Canadian Medical Association Journal = Journal De L’association Medicale Canadienne*, *192*(6), E136–E141. 10.1503/cmaj.190434 PMC7012622 32041697

[CIT4344944] Abd Rahman, A. A., & Abdul Razak, F. H. (2019). Social media addiction towards young adults emotion. Journal of Media and Information Warfare (JMIW), 12(2), 1–15.

[CIT0001] Agnew, H. (2017). *France takes phones away from tech-addicted teenagers*. Retrieved April 09, 2024 https://www.ft.com/content/fdc1944e-e1a6-11e7-8f9f-de1c2175f5ce

[CIT0002] Ainsworth, B. E., & Der Ananian, C. (2020). Physical activity promotion. In *Handbook of Sport Psychology* (pp. 773–794). Wiley Online Library. 10.1002/9781119568124.ch37

[CIT0003] Alexander, S. (2022). A uniquely Australian approach: A thematic analysis of the normative foundations of Australia’s approach to the regulation of the internet. *Adelaide Law Review*, *43*, 345.

[CIT0004] Andreassen, C. S. (2015). Online social network site addiction: A comprehensive review. *Current Addiction Reports*, *2*(2), 175–184. 10.1007/s40429-015-0056-9

[CIT0005] Andreassen, C. S., Pallesen, S., & Griffiths, M. D. (2017). The relationship between addictive use of social media, narcissism, and self-esteem: Findings from a large national survey. *Addictive Behaviors*, *64*, 287–293. 10.1016/j.addbeh.2016.03.006 27072491

[CIT0006] Bail, C. A., Argyle, L. P., Brown, T. W., Bumpus, J. P., Chen, H., Fallin Hunzaker, M. B., Lee, J., Mann, M., Merhout, F., & Volfovsky, A. (2018). Exposure to opposing views on social media can increase political polarization. *Proceedings of the National Academy of Sciences of the United States of America*, *115*(37), 9216–9221. 10.1073/pnas.1804840115 30154168 PMC6140520

[CIT0007] Bányai, F., Zsila, Á., Király, O., Maraz, A., Elekes, Z., Griffiths, M. D., Schou Andreassen, C., & Demetrovics, Z. (2017). Problematic social media use: Results from a large-scale nationally representative adolescent sample. *PloS One*, *12*(1), e0169839. 10.1371/journal.pone.0169839 28068404 PMC5222338

[CIT0008] Barkley, T. (2024). *What should Policymakers do about social media and minors?* The Center for Growth and Opportunity.

[CIT0009] Barrios, C. H., & Mano, M. S. (2021). Is independent clinical research possible in low-and middle-income countries? A roadmap to address persistent and new barriers and challenges. In *American Society of Clinical Oncology Educational Book. American Society of Clinical Oncology. Annual Meeting* (vol. 41, pp. 1–10). ASCO Publications. 10.1200/EDBK_321335 33830826

[CIT0011] BBC iReporter. (n.d). Retrieved April 10, 2024 https://www.bbc.co.uk/news/resources/idt-8760dd58-84f9-4c98-ade2-590562670096

[CIT0012] Bozdag, E., & Van Den Hoven, J. (2015). Breaking the filter bubble: Democracy and design. *Ethics and Information Technology*, *17*(4), 249–265. 10.1007/s10676-015-9380

[CIT0013] Brady, W. J., Wills, J. A., Jost, J. T., Tucker, J. A., & Van Bavel, J. J. (2017). Emotion shapes the diffusion of moralized content in social networks. *Proceedings of the National Academy of Sciences of the United States of America*, *114*(28), 7313–7318. 10.1073/pnas.1618923114 28652356 PMC5514704

[CIT0014] Brand, M., Laier, C., & Young, K. S. (2014). Internet addiction: Coping styles, expectancies, and treatment implications. *Frontiers in Psychology*, *5*, 1256. 10.3389/fpsyg.2014.01256 25426088 PMC4227484

[CIT0015] Brand, M., Potenza, M. N., & Stark, R. (2022). Theoretical models of types of problematic usage of the Internet: When theorists meet therapists. *Current Opinion in Behavioral Sciences*, *45*, 101119. 10.1016/j.cobeha.2022.101119

[CIT0016] Brooks, S. K., Webster, R. K., Smith, L. E., Woodland, L., Wessely, S., Greenberg, N., & Rubin, G. J. (2020). “The psychological impact of quarantine and how to reduce it: Rapid review of the evidence. *Lancet (London, England)*, *395*(10227), 912–920. 10.1016/S0140-6736(20)30460-8 32112714 PMC7158942

[CIT0017] Cataldo, I., Billieux, J., Esposito, G., & Corazza, O. (2022). Assessing problematic use of social media: Where do we stand and what can be improved? *Current Opinion in Behavioral Sciences*, *45*, 101145. 10.1016/j.cobeha.2022.101145

[CIT0018] Cherry, K. (2022). *What is social Reinforcement?* Retrieved January 19, 2024. https://www.verywellmind.com/what-is-social-reinforcement-2795881

[CIT0019] Coyne, S. M. (2023). *The Unintended Consequences of Banning Social Media-Severely limiting teen access to social media might do more harm than good*. Retrieved April 09, 2024. https://www.psychologytoday.com/us/blog/the-right-media-mindset/202302/the-unintended-consequences-of-banning-social-media

[CIT0020] Daniel, F. (2022). *Marketing Monday- Scrolling the Statue of Liberty*. Retrieved February 27, 2025 https://www.opticaljournal.com/marketing-monday-scrolling-the-statue-of-liberty

[CIT0021] Daniels, M., Sharma, M., & Batra, K. (2021). Social media, stress and sleep deprivation: A triple “S” among adolescents. *Journal of Health and Social Sciences*, *6*(2), 159. 10.19204/2021/sclm3

[CIT34342021] David, G. (2015). Why is Facebook banned in China? Retrieved from: https://www.quora.com/Why-is?Facebook-banned-in-China-1 Accessed 09 April 2024.

[CIT0022] Del Vicario, M., Scala, A., Caldarelli, G., Eugene Stanley, H., & Quattrociocchi, W. (2017). Modeling confirmation bias and polarization. *Scientific Reports*, *7*(1), 40391. 10.1038/srep40391 28074874 PMC5225437

[CIT0023] Donabauer, G., Ognibene, D., Kruschwitz, U., Hernández-Leo, D., Fulantelli, G., & Hoppe, H. U. (2022). The “Courage Companion”–An AI-Supported Environment for Training Teenagers in Handling Social Media Critically and Responsibly. In *Higher Education Learning Methodologies and Technologies Online: 4th International Conferen*ce, HELMeTO. September 21–232022, Revised Selected Papers (p. 395). Springer Nature. 2023

[CIT0024] Erzikova, E., Waters, R., & Bocharsky, K. (2018). Media catching: A conceptual framework for understanding strategic mediatization in public relations? *International Journal of Strategic Communication*, *12*(2), 145–159. 10.1080/1553118X.2018.1424713

[CIT0025] Esmaeili Rad, M., & Ahmadi, F. (2018). A new method to measure and decrease the online social networking addiction. *Asia-Pacific Psychiatry: official Journal of the Pacific Rim College of Psychiatrists*, *10*(4), e12330. 10.1111/appy.12330 30175904

[CIT0026] European Commission. (2019). Retrieved January 19, 2024 https://ec. europa. eu/eurostat/statistics-explained/index. php. Waste_statistics# Total_waste_generation

[CIT0027] Festl, R. (2021). Social media literacy & adolescent social online behavior in Germany. *Journal of Children and Media*, *15*(2), 249–271. 10.1080/17482798.2020.1770110

[CIT0028] Fulantelli, G., Taibi, D., Scifo, L., Schwarze, V., & Eimler, S. C. (2022). Cyberbullying and cyberhate as two interlinked instances of cyber-aggression in adolescence: A systematic review. *Frontiers in Psychology*, *13*, 909299. 10.3389/fpsyg.2022.909299 35712182 PMC9196243

[CIT0030] Gammon, M. A., & White, J. (2011). (Social) media literacy: Challenges and opportunities for higher education. *Educating Educators with Social Media*, 1, 329–345. 10.1108/S2044-9968(2011)0000001019

[CIT0031] Gao, J., Zheng, P., Jia, Y., Chen, H., Mao, Y., Chen, S., Wang, Y., Fu, H., & Dai, J. (2020). Mental health problems and social media exposure during COVID-19 outbreak. *PloS One*, *15*(4), e0231924. 10.1371/journal.pone.0231924 32298385 PMC7162477

[CIT0032] Gillani, N., Yuan, A., Saveski, M., Vosoughi, S., & Roy, D. (2018). *Me, my echo chamber, and I: Introspection on social media polarization* [Paper presentation]. Proceedings of the 2018 World Wide Web Conference (pp. 823–831). 10.1145/3178876.3186130

[CIT0033] Glüer, M., & Lohaus, A. (2018). Parental and child assessment of parental media education strategies and their connection with children’s Internet use competence. *Practice of Child Psychology and Child Psychiatry*, *2*(67), 181–203. 10.13109/prkk.2018.67.2.181

[CIT0034] Grant, J. E., Potenza, M. N., Weinstein, A., & Gorelick, D. A. (2010). Introduction to behavioral addictions. *The American Journal of Drug and Alcohol Abuse*, *36*(5), 233–241. 10.3109/00952990.2010.491884 20560821 PMC3164585

[CIT0035] Griffiths, M. (2005). A ‘components’ model of addiction within a biopsychosocial framework. *Journal of Substance Use*, *10*(4), 191–197. 10.1080/14659890500114359

[CIT0036] Hazleton, V. (1992). Toward a systems theory of public relations. *Ist Public Relations eine Wissenschaft? Eine Einführung.* 33–45. 10.1007/978-3-322-85772-9_3

[CIT0037] Hazleton, V. (1993). Symbolic resources processes in the development and use of symbolic resources. In *Image und PR: Kann Image Gegenstand einer Public Relations-Wissenschaft sein?* (pp. 87–100). 10.1007/978-3-322-85729-3_6

[CIT0038] Hess, U., & Fischer, A. (2013). Emotional mimicry as social regulation. *Personality and Social Psychology Review: An Official Journal of the Society for Personality and Social Psychology, Inc*, *17*(2), 142–157. 10.1177/1088868312472607 23348982

[CIT0039] Hinde, S., McKenna, C., Whyte, S., Peake, M. D., Callister, M. E. J., Rogers, T., & Sculpher, M. (2015). Modelling the cost-effectiveness of public awareness campaigns for the early detection of non-small-cell lung cancer. *British Journal of Cancer*, *113*(1), 135–141. 10.1038/bjc.2015.167 26010412 PMC4647547

[CIT5878765] Joa, C. Y., Kim, K., & Ha, L. (2018). What Makes People Watch Online In-Stream Video Advertisements?. *Journal of Interactive Advertising*, *18*(1), 1–14. 10.1080/15252019.2018.1437853

[CIT0040] Lee, R. S. C., Hoppenbrouwers, S., & Franken, I. (2019). A systematic meta-review of impulsivity and compulsivity in addictive behaviors. *Neuropsychology Review*, *29*(1), 14–26. 10.1007/s11065-019-09402-x 30927147

[CIT0041] Lee, Y., Jin Jeon, Y., Kang, S., Shin, J. I., Jung, Y.-C., & Jung, S. J. (2022). Social media use and mental health during the COVID-19 pandemic in young adults: A meta-analysis of 14 cross-sectional studies. *BMC Public Health*, *22*(1), 995. 10.1186/s12889-022-13409-0 35581597 PMC9112239

[CIT0042] Lenhart, A. (2015). *Teens, social media & technology overview 2015*.

[CIT3564728] Li, YAN., & Ranieri, M. (2010). Are ‘digital natives’ really digitally competent?—A study on Chinese teenagers. *British Journal of Educational Technology*, *41*(6), 1029–1042. 10.1111/j.1467-8535.2009.01053.x

[CIT0043] Liu, S., Yang, L., Zhang, C., Xiang, Y.-T., Liu, Z., Hu, S., & Zhang, B. (2020). Online mental health services in China during the COVID-19 outbreak. *The Lancet. Psychiatry*, *7*(4), e17–e18. 10.1016/S2215-0366(20)30077-8 32085841 PMC7129099

[CIT0044] Livingstone, S., Ólafsson, K., Helsper, E. J., Lupiáñez-Villanueva, F., Veltri, G. A., & Folkvord, F. (2017). Maximizing opportunities and minimizing risks for children online: The role of digital skills in emerging strategies of parental mediation. *Journal of Communication*, *67*(1), 82–105. 10.1111/jcom.12277

[CIT0045] Lundahl, O. (2021). Media framing of social media addiction in the UK and the US. *International Journal of Consumer Studies*, *45*(5), 1103–1116. 10.1111/ijcs.12636

[CIT722939588] M, A., McAlaney, B., Skinner, J., Pleya, T., Ali, M., & R. (2020). Defining digital addiction: Key features from the literature. *Psihologija*, *53*(3), 237–253. 10.2298/PSI191029017A

[CIT0046] Marchant, A., Hawton, K., Stewart, A., Montgomery, P., Singaravelu, V., Lloyd, K., Purdy, N., Daine, K., & John, A. (2017). A systematic review of the relationship between internet use, self-harm and suicidal behaviour in young people: The good, the bad and the unknown. *PloS One*, *12*(8), e0181722. 10.1371/journal.pone.0181722 28813437 PMC5558917

[CIT0047] Meola, C. (2020). Helping Aussie women online: A discourse analysis of the Australian eSafety Commissioner website. In *Social media in legal practice* (pp. 130–145). Routledge. 10.4324/9780429346088-9

[CIT0048] Michie, S., Atkins, L., & West, R. (2014). The behaviour change wheel. *A Guide to Designing Interventions*, *1*, 1003–1010.

[CIT0049] Murgia, M. (2015). *Brazil removes ban on WhatsApp after protests*. http://www.telegraph.co.uk/technology/social-media/12055400/Brazil-bans-WhatsApp-for-48- hours.htm

[CIT0050] Nevard, I., Green, C., Bell, V., Gellatly, J., Brooks, H., & Bee, P. (2021). Conceptualising the social networks of vulnerable children and young people: A systematic review and narrative synthesis. *Social Psychiatry and Psychiatric Epidemiology*, *56*(2), 169–182. 10.1007/s00127-020-01968-9 33140120 PMC7870613

[CIT0051] Oxford Dictionary. (2000). *‘Intrinsic’ definition.* Oxford advanced learner’s dictionary. Retrieved from Oxford Learner Dictionaries.

[CIT0052] Paakkari, L., Tynjälä, J., Lahti, H., Ojala, K., & Lyyra, N. (2021). Problematic social media use and health among adolescents. *International Journal of Environmental Research and Public Health*, *18*(4), 1885. 10.3390/ijerph18041885 33672074 PMC7919645

[CIT0053] Pagoto, S., Waring, M. E., & Xu, R. (2019). A call for a public health agenda for social media research. *Journal of Medical Internet Research*, *21*(12), e16661. 10.2196/16661 31855185 PMC6940852

[CIT0054] Paid Media Pros. (2023). *Facebook Non-Skippable Ads*. https://www.youtube.com/watch?v=_4OO9q1oYfU

[CIT0055] Pellegrino, A., Stasi, A., & Bhatiasevi, V. (2022). Research trends in social media addiction and problematic social media use: A bibliometric analysis. *Frontiers in Psychiatry*, *13*, 1017506. 10.3389/fpsyt.2022.1017506 36458122 PMC9707397

[CIT0056] Postmes, T., & Spears, R. (1998). Deindividuation and antinormative behavior: A meta-analysis. *Psychological Bulletin*, *123*(3), 238–259. 10.1037/0033-2909.123.3.238

[CIT0057] Rahim, S., & Amir, A. (2019). Mohd Nasir Markom, and Syed Agil Alsagoff. “The roles of public relations in an environmental awareness campaign: A case study of SWM environment SDN BHD. *Jurnal Kemanusiaan*, 17, 32-42.

[CIT0058] Rahman, A., Amiera, A., & Razak, F. H. A. (2019). Social media addiction towards young adults emotion. *Journal of Media and Information Warfare (JMIW)*, *12*(2), 1–15. 10.1503/cmaj.190434

[CIT0059] Ritchie, H. (2024). *Australia approves social media ban on under-16s*. https://www.bbc.co.uk/news/articles/c89vjj0lxx9o

[CIT0060] Rudy, L. J. (2023). *When is Impulsivity a Problem?* https://www.verywellhealth.com/impulsivity-5270462

[CIT0061] Schier, S. E., & Eberly, T. E. (2016). *Polarized: The rise of ideology in American politics*. Rowman & Littlefield.

[CIT0062] Science and Technology Committee. (2019). *Impact of social media and screen-use on young people’s health.* https://publications.parliament.uk/pa/cm201719/cmselect/cmsctech/822/822.pdf

[CIT0063] Scottish Legal News. (2023). *France: New Parental Consent Law to regulate Children’s Social media use*. https://www.scottishlegal.com/articles/france-new-parental-consent-law-to-regulate-childrens-social-media-use

[CIT0064] Seymour, J. (2018). The impact of public health awareness campaigns on the awareness and quality of palliative care. *Journal of Palliative Medicine*, *21*(S1), S30–S36. 10.1089/jpm.2017.0391 29283867 PMC5733664

[CIT0065] Singh, S., Dixit, A., & Joshi, G. (2020). Is compulsive social media use amid COVID-19 pandemic addictive behavior or coping mechanism? *Asian Journal of Psychiatry*, *54*, 102290. 10.1016/j.ajp.2020.102290 32659658 PMC7338858

[CIT0066] Stanley, T. (2018). *Social media has become a public health issue—And it’s killing us all*. https://www.telegraph.co.uk/ news/2018/04/23/social-media-has-become-public-health-issue -killing-us/.

[CIT0067] Steinert, J. I., Atika Nyarige, D., Jacobi, M., Kuhnt, J., & Kaplan, L. (2021). A systematic review on ethical challenges of ‘field’research in low-income and middle-income countries: Respect, justice and beneficence for research staff? *BMJ Global Health*, *6*(7), e005380. 10.1136/bmjgh-2021-005380 PMC8292801 34285041

[CIT0068] Stewart, J. M. (2013*). Network Security, Firewalls and VPNs (неопр.).*

[CIT0069] Strieter, L., Laddu, D. R., Sainsbury, J., & Arena, R. (2019). The importance of school-based healthy living initiatives: Introducing the Health and Wellness Academy concept. *Progress in Cardiovascular Diseases*, *62*(1), 68–73. 10.1016/j.pcad.2018.08.005 30236752

[CIT0070] Taibi, D., Börsting, J., Hoppe, U., Ognibene, D., Hernández-Leo, D., Eimler, S. C., & Kruschwitz, U. (2022). The role of educational interventions in facing social media threats: overarching principles of the courage project. In *International workshop on higher education learning methodologies and technologies online* (pp. 315–329). Springer Nature Switzerland. 10.1007/978-3-031-29800-4_25

[CIT0071] Tandon, R. (2020). The COVID-19 pandemic, personal reflections on editorial responsibility. *Asian Journal of Psychiatry*, *50*, 102100. 10.1016/j.ajp.2020.102100 32354694 PMC7165287

[CIT0072] The Hoops Geek. (2022). The Average Height of NBA Players from 1952-2022. Retrieved February 27, 2025 https://www.thehoopsgeek.com/average-nba-height/

[CIT0073] Tuncay, N. (2018). *Social Media Ban: Virtually Social or Physically Social*. Çözüm Educational Publishing.

[CIT0074] Ureña, R., Kou, G., Dong, Y., Chiclana, F., & Herrera-Viedma, E. (2019). A review on trust propagation and opinion dynamics in social networks and group decision making frameworks. *Information Sciences*, *478*, 461–475. 10.1016/j.ins.2018.11.037

[CIT0075] Webb, H., Burnap, P., Procter, R., Rana, O., Stahl, B. C., Williams, M., Housley, W., Edwards, A., & Jirotka, M. (2016). Digital wildfires: Propagation, verification, regulation, and responsible innovation. *ACM Transactions on Information Systems*, *34*(3), 1–23.) 10.1145/2893478

[CIT0076] Werder, K. P. (2005). An empirical analysis of the influence of perceived attributes of publics on public relations strategy use and effectiveness. *Journal of Public Relations Research*, *17*(3), 217–266. 10.1207/s1532754xjprr1703_2

[CIT0077] Werder, K. P., & Holtzhausen, D. (2009). An analysis of the influence of public relations department leadership style on public relations strategy use and effectiveness. *Journal of Public Relations Research*, *21*(4), 404–427. 10.1080/10627260902966391

